# The Design and Application of Simplified Insole-Based Prototypes with Plantar Pressure Measurement for Fast Screening of Flat-Foot

**DOI:** 10.3390/s18113617

**Published:** 2018-10-25

**Authors:** Wei-Chun Hsu, Tommy Sugiarto, Jun-Wen Chen, Yi-Jia Lin

**Affiliations:** 1 Graduate Institute of Biomedical Engineering, National Taiwan University of Science and Technology, Taipei 10607, Taiwan; d10622817@mail.ntust.edu.tw (T.S.); m10523102@mail.ntust.edu.tw (J.-W.C.); jiajia527@gmail.com (Y.-J.L.); 2 Graduate Institute of Applied Science and Technology, National Taiwan University of Science and Technology, Taipei City 10607, Taiwan; 3 Department of Biomedical Engineering, National Defense Medical Center, Taipei 11490, Taiwan; 4 Division of Embedded System and SoC Technology, System Integration and Application Department, Information and Communication Research Laboratory, Industrial Technology Research Institute, Hsinchu 31057, Taiwan

**Keywords:** plantar pressure, flat foot, insoles, force sensors, arch index

## Abstract

This study aimed to find the correlation between conventional Arch Index (AI) measurements and our prototype of a simplified insole-based plantar pressure measurement system and to find out the effective plantar pressure sensor position. Twenty-one subjects participated in this study, which was divided into two groups: 10 subjects with flatfoot and 11 subjects with normal foot. Five force sensitive resistance sensors were used on this prototype using Arduino as the data acquisition device. Two types of trials, namely static and dynamic, were conducted to validate our system against the ink-type AI measurement as a golden standard. The results showed that in the static trial, there was a high linear correlation with the medial arch sensor configuration, while in the dynamic trial, there was a high linear correlation in the medial arch sensor configuration and sensor 5 configuration. This study showed that both static and dynamic tests using the self-developed device could effectively determine most of the flatfoot subjects and suggests that in the future, it can be applied in clinical applications because of its advantages when compared to the expensive-high tech graphic input board and conventional tools, like ink-type based measurements.

## 1. Introduction

The arch of the foot helps in absorbing and transferring energy when walking or running. Thus, an abnormal arch, such as flatfoot, often leads to an abnormal gait and running pattern. Flatfoot can be divided into flexible flatfoot and rigid flatfoot, with the former, also called functional flatfoot, meaning that the arch is flat when the foot is weight bearing, or that the arch is evident when the toes of the foot point outwards while the subjects stand in non-weight bearing or through the toe-raising of the Jack Test proposed in 1953 [1]. While the latter, also called structural flatfoot, refers to when the arch is not present, whether in weight bearing status or not, and the subtalar joint movement is poor. Ninety-five percent of patients with flatfoot are flexible flatfoot [2]. Patients with rigid flatfoot often suffer from pain or potentially more severe pathological problems, such as tarsal coalition or neuromuscular flatfoot [3], etc. Rigid flatfoot mostly requires surgery rather than being treated by traditional treatments if patients are in pain [2].

Currently, the methods for judging flatfoot have been mainly focused on the medial longitude arch (MLA), and common methods include the traditional arch height, the navicular drop test [4,5], the footprint index classification [6,7,8,9], and X-ray [10]. The arch height can be quantified by the difference between the highest point of the soft tissue on the edge of the medial arch and the ground [11]. The navicular drop test (NDT) was first described as a means of representing the height change of the navicular bone under-weight bearing and non-weight bearing [5], with a moderate to good intra-reproducibility of the measurement [12]. The level of navicular drop refers to the height difference of the navicular tubercle in the standing and sitting position, and has been reported to be significantly correlated with the degree of the pronation of the foot of a runner, and has been used for judging flatfoot [4].

The footprint index classification is commonly used in research and the traditional method is mostly conducted with an ink-type instrument, which has advantages over other clinical tests, with its high reproducibility [13,14]. More recently, the plantar pressure system or the plantar scanning system has been used to measure, calculate, and determine the footprint. Although the footprints are measured in different ways, the method of judging flatfoot is the same. There are different calculations in terms of footprint classification, where the common arch index (AI) is obtained while the subjects stand evenly on the ink board one foot at a time [6]. The footprints are divided into three equal areas based on the foot length of the footprints to calculate the AI of the subjects and also quantify the severity of flatfoot [15]. The AI is highly reliable and correlates with X-ray measurements, so has been used for determining flatfoot in research [13]. The judgment for the type of arch relies on X-rays in hospital, which is reliable, but time-consuming and expensive.

Plantar pressure, also known as the pressure between the foot and the support surface, can be calculated by the force of the vertical contact divided by the contact area. Obtaining the plantar pressure information is helpful for assessing clinical problems. For example, the peak plantar pressure of the forefoot and hindfoot as well as the value of the peak forefoot pressure divided by the peak hindfoot pressure of severe diabetic neuropathic patients were significantly higher than that of patients with mild ulceration or with no history of ulceration. This was determined to be the possible reason as to why diabetic patients easily face the risk of foot ulceration. Therefore, plantar pressure can be regarded as a parameter to prevent foot ulceration for diabetic patients [16].

The average pressure has also been studied under 10 anatomical areas, including the medial and lateral heel area, the medial and lateral mid-foot area, the heads of the first, second, and fifth metatarsal area, the great toe, the head of the second metatarsal area, and the lateral metatarsal head area [15]. However, the placement of different insoles could effectively affect these pressure indices [17]. Thus, recent studies have addressed how to simplify the number of sensors in the plantar pressure insole or to place sensors at relatively important positions based on applications to reduce the development cost as well as provide relatively accurate data for launching products in the market to consumers [18]. Reduction of the spatial and temporal image resolution by placing pressure sensors at only seven locations, namely the heel, lateral midfoot, lateral forefoot, great toe, head of the first metatarsal, center midfoot, and center forefoot has been used for measuring and evaluating plantar pressure during activities of daily living for further gait analysis and interpretation of pathological foot anatomy.

A cluster type of sensor placement, being three on the metatarsal and three on the heels with a total of only six points, was developed, with the aim of further comparing the results between those obtained from state-of-the-art equipment with those obtained from sensors on the insole [19]. By placing sensors on the insole, these cost-effective and efficient wearable devices allow the monitoring and analysis of the gait anywhere from large clinics to an individual’s home, and can be applied to produce graphics similar to those measured from the force-plate [20,21], where fourteen sensors of the Force Sensitive Resistance (FSR) Model 402 (Interlink Electronics, Camarillo, CA, USA) were placed on the forefoot, midfoot, and hindfoot to measure plantar pressure. In 2016, Liang et al. also developed an insole-based plantar pressure measurement with a fiber-optic sensing system where six fiber-optic sensors were used and embedded in silicone rubber. The results showed that the system could successfully identify the four different foot types and their developed-system could reach a Pearson correlation coefficient of 0.671 compared to the *i-Step P1000* plantar pressure plate [22].

It has been suggested that the traditional visual judgment of flatfoot was too subjective, and that flatfoot should be detected by objective and accurate optical or electronic systems [23]. This study suggested how to design the insole-based plantar pressure instrument for flatfoot. However, it lacked practical clinical verification. However, there are few insole-based plantar pressure measurement systems for flatfoot, therefore, methods of measuring flatfoot with a simplified framework of software, hardware, and design to develop a low-cost wearable insole-based instrument that allows for the measurement of plantar pressure to quickly and successfully screen flatfoot and normal foot during both static and dynamic conditions are of clinical needs.

Thus, the aims of this study were first to perform the correlation between traditional AI and the value of each point of the self-made insole-based sensor to find the effective plantar pressure sensor position and to compare whether there were significant differences between the sensors at specific positions of flatfoot and normal foot from the data of 21 young subjects.

## 2. Materials and Methods

### 2.1. Subject

Twenty-one healthy subjects with shoes sized between US9/Euro 42 and US10/Euro 43–44 participated in this study. Ten subjects with flatfoot and 11 with normal foot were classified by ink-type AI. Healthy subjects had no history of affecting postural stability, or gait in the past 12 months, and/or cognitive impairment. This experiment was approved by local research ethics. Subjects who injured their lower extremity six months ago before participation and still suffered from pain, who had surgery on their hip joints, knee joints, and ankle joints of the lower extremity, and who could not walk or stand for the pain in the lower extremity or back were excluded from the study. The subject’s characteristics, including their Body Mass Index (BMI) and ratio of foot length and navicular-to-toe length, are shown in Table 1.

### 2.2. Equipment

#### 2.2.1. Ink-Type Footprint Printer

According to the method proposed by Cavanagh in 1987, the subjects stood statically with even force on the ink-type footprint printer, then the subjects’ footprints were obtained on the paper by the ink [6].

**Figure 1:**
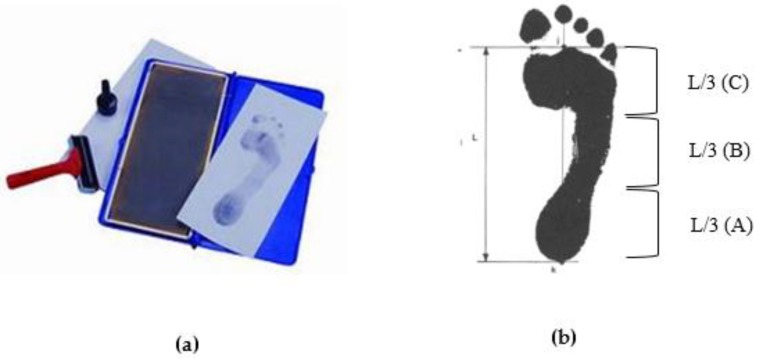
Ink-type footprint printer (**a**,**b**) the method of arch index calculation [6].

The measured footprint was drawn from the second metatarsal bone to the center of the heel, which is called the foot axis. Next, the foot axis was divided vertically into three equal parts, namely the forefoot (A), the midfoot (B), and the hindfoot (C) (Figure 1). The AI was calculated by the midfoot area (B) being divided by the full foot area (A + B + C). If the AI was less than or equal to 0.21, it was regarded as high arch; if the AI was between 0.21 and 0.26, it was regarded as normal foot; if the AI was greater than 0.26, it was regarded as flatfoot [6].

#### 2.2.2. Self-Made Simplified Instrument

**Figure 2:**
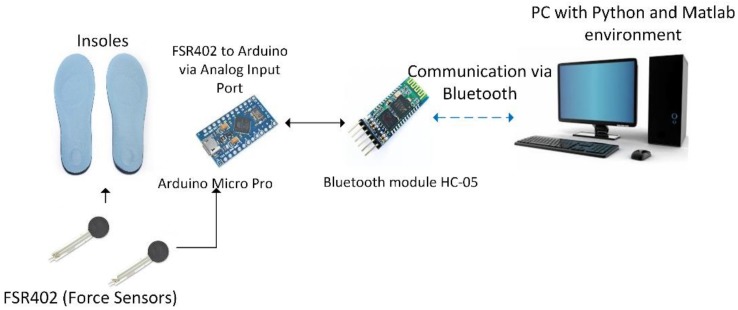
The hardware system diagram of the FSR-based insoles for measuring the flatfoot and normal foot.

Combining the clinical and electronics circuit design ability, we designed a simplified self-made instrument to effectively measure flatfoot and normal foot. The hardware system design diagram is shown in Figure 2. An Arduino Micro Pro was used combined with the Force FSR placed on insoles to measure the foot plantar pressure and transmitted the data to a personal computer by Bluetooth connection.

#### 2.2.3. Hardware System Design

The entire hardware system was designed with an Arduino Mirco Pro and the hardware system diagram is shown in Figure 2. The Arduino Micro Pro used in this study was based on ATmega32u4 as its processor. The analog input channel connected five FSR to sense the external pressure, and the data from the FSR were transmitted to a PC with the Python environment via Bluetooth connection (HC-05 Bluetooth module). This system used a rechargeable lithium battery as the power source, thus the TP-4056 module was used as a battery charger and protection module.

**Figure 3:**
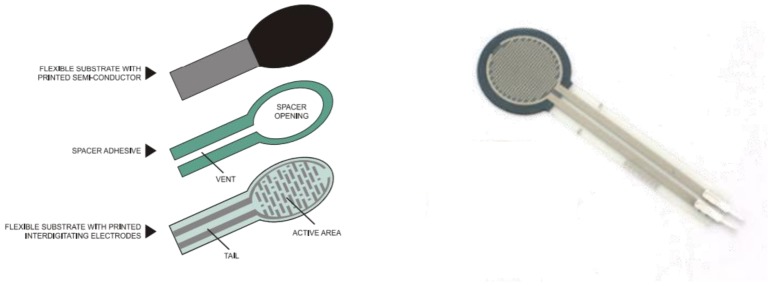
FSR-Interlink402 sensors (**right**) and its detailed configuration (**left**).

The FSR sensor used in this study was an Interlink-402 that could measure the force values up to 100 g–10 kg. The FSR is composed of a flexible substrate with a printed semi-conductor, flexible substrate with interdigitating electrode, and spacer adhesive. FSR is quite cheap and easily used with a small size (the diameter of the sensing area is 12.7 mm) and thin thickness (thickness of 0.45). FSR is not as accurate as the load cell or the strain gauge, however, it can reduce measurement errors or use relative comparisons through individual calibration [24]. In this study, the calibration was done before the FRS were put on the insoles, with the calibration procedure independently applied to each sensor. Data processing for this calibration procedure was done with Matlab 2017b (MathWorks Inc., Natick, MA, USA). The Interlink402-FSR is shown in Figure 3.

Special attention should be paid to the so-called force repeatability in the parameters of force sensing components as force repeatability indicates that the resistance values may change between ±15%~±25% with the same force in different sensors. As a result, each sensing component should be individually calibrated before use. The nickel-clad copper precision weight of the electronic scale weight was used to calibrate each FSR. Referring to the static calibration described by Flórez in 2010, the precision weight is regarded as a fixed force. In the calibration process, weights of different amounts were placed on the platform, with the force evenly applied on the FSR, before finally, the resistances were recorded under different forces by the algorithm [24].

#### 2.2.4. Sensor Placement Design

**Figure 4:**
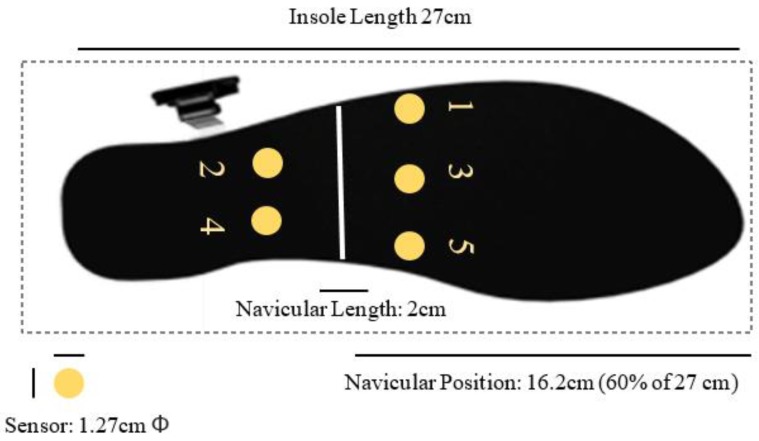
Sensor placement and numbering on the insoles.

As discussed in the literature review, flatfoot is judged mainly by whether the MLA (medial longitude arch) drops. From the perspective of plantar anatomy, the drop of the navicular bone is the main cause, thus the regular position of the navicular bone should be referred to before designing the sensor placement. According to the statistics of the experiment, the position of the navicular bone falls between 60% and 65% of the whole foot, and the length of an adult’s navicular bone ranges between 1–2 cm. Therefore, we decided to set 60% as the position of the navicular bone where the sensors were placed with the distance of 1 cm on the front and back. The sensors were placed in an array and numbered from 1 to 5 from the medial to the lateral, respectively. The illustration of the sensor placement and numbering is shown in Figure 4.

#### 2.2.5. Experimental Procedure

• Measurement of the ratio of the foot length and navicular length

**Figure 5:**
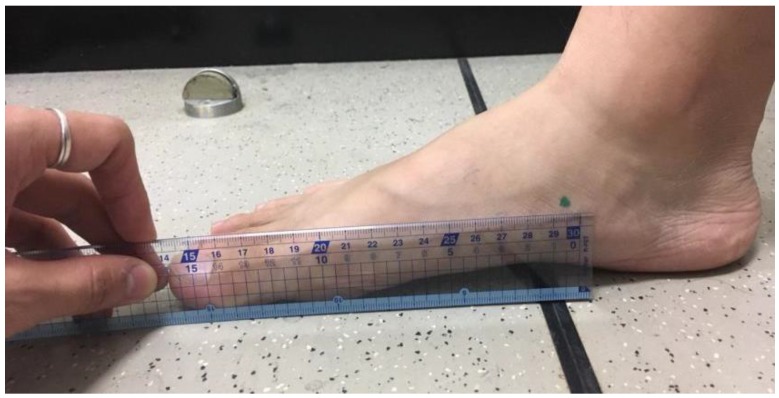
Illustration of the measurement procedure of the navicular length.

In this experiment, the foot length was measured with a foot gauge. The subjects were required to stand evenly, placing one foot on the foot gauge. The heel of the foot was measured to the front position of the foot (great toe or index toe). The protruding position of the navicular bone was found and marked by palpation, then the length from the navicular bone to the front position of the foot was measured by a ruler. After recording, the value was divided by the foot length to obtain the position ratio of the corresponding navicular bone (Figure 5).

• Ink-type footprint measurement

**Figure 6:**
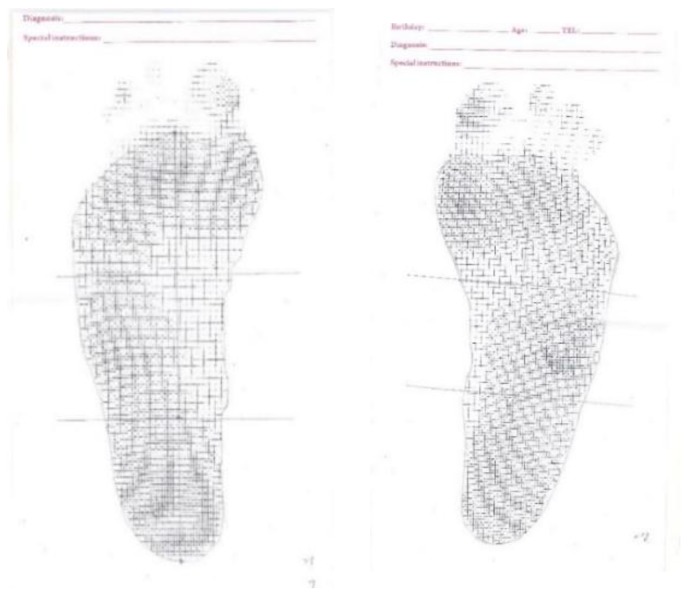
Typical example of ink-type footprint diagram.

As an index of determining the foot type in the current study, the measurement and calculation of the ink-type footprint refers to that mentioned in the paper by Cavanagh in 1987 [6,15], where the subjects stand statically with one foot on the ink-type footprint printer that prints the footprint of the subjects. The measurement needs to be repeated if swaying or over-imprint occurs (such as standing with one foot) (Figure 6).

#### 2.2.6. Static Standing Insole-Type Plantar Pressure Measurement

**Figure 7:**
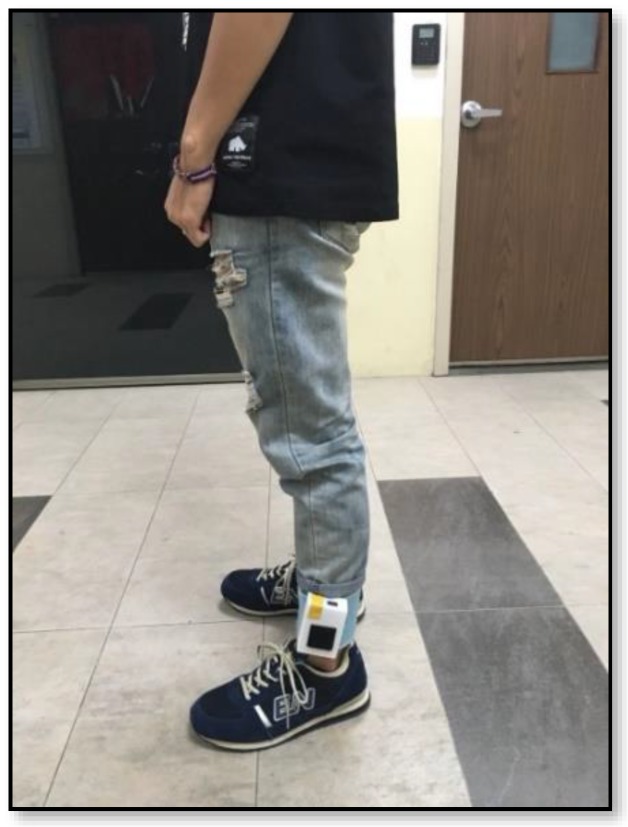
Static standing plantar pressure measurement position for the subject, with the insole-plantar pressure device inserted in the shoes.

Based on the methods proposed by Cavanagh in 1987, the movements were designed to test whether the self-made insole-type plantar was able to determine the subject’s foot type. The subjects stood evenly on the insole-type plantar pressure instrument, and the designed insole-type plantar pressure instruments were placed in a normal shoe that served as the test shoe in the current study. Before using this normal shoe, all the additional support for the arch had already been removed to ensure that there was no other arch support, which may bias the result. On this statically standing position, the subjects had to look straight ahead with their hands at their sides for five seconds with the sample rate of 100 Hz (as shown in Figure 7).

#### 2.2.7. Dynamic Insole-Type Plantar Pressure Measurement

The subjects were asked to walk in a dynamic trial while wearing the self-made insole-type plantar pressure device. The stance phase of the gaits was calculated the period where the heel makes contact with the ground to when the soles are off the ground. One sensor was placed on the heel to define the heel-strike time while the other one was placed on the forefoot to detect the toe-off time. The stance phase during the dynamic trials was started at the heel-strike and ended at the toe-off.

Each subject needed to complete three successful trials with their preferred speed. The subject’s preferred speed was chosen to avoid an un-natural walking style of the subject if they walked at the specified chosen speed. The trial had to be recollected if there was any instability or if the subject did not look straight ahead.

### 2.3. Data Analysis

The force data from the first to fifth sensors in the static and dynamic trial were calculated, respectively. In terms of static standing, the average of the force of static standing for five seconds was divided by the body weight. During analysis, this study calculated several indices. First, the average normalized force of each sensor for five seconds. Second, this study viewed the third sensor as the center column of the arch, which divided the arch into the medial arch and lateral arch and the third sensor also contributed both the medial side and lateral, the medial arch (the sum of the average force of the fifth sensor, the fourth sensor, and the third sensor was divided by two), and the lateral arch (the sum of the average force of the first sensor, the second sensor, and the third was divided by two). Third, the first three sensors (the sum of the first, the third, and the fifth) and the five sensors were calculated.

In terms of the dynamic trial, three successful gait trials were collected, and the maximum value of each gait of each sensor, the maximum of the sum of the medial arch in Equation (1), the average force of the lateral arch in Equation (2), the maximum of the sum of the first three sensors, and the maximum force of five sensors were calculated.

(1) $$Max\;\left(Sensor\;5+Sensor\;4+\frac{Sensor\;3}{2}\right)$$

(2) $$Max\;\left(Sensor\;1+Sensor\;2+\frac{Sensor\;3}{2}\right)$$

All the force data calculated in this paper were normalized by their own body weight (kgf/kg) to minimize the effect of the individual difference.

### 2.4. Statistical Analysis

Statistical analysis was performed to obtain the Pearson r correlation coefficient by linear regression between the calculated data and the ink-type AI. A correlation coefficient falling between 0.7–0.99 was categorized as highly correlated, while 0.4–0.6 was categorized as a moderate correlation, 0.1–0.39 was categorized as a low correlation, and 0–0.1 was categorized as extremely uncorrelated.

For the comparison between groups, the Mann–Whitney U Test was used for the between-group comparison of pressure parameters between flatfoot and normal foot type groups with the significance level set at alpha = 0.05.

The reliability analysis for examining the reliability of the proposed system when several measurements on the same subject was performed with the Intraclass Correlation Coefficient (ICC) with a two-way mixed model (ICC _3,1_) and Cronbach’s alpha coefficient.

## 3. Results

The experimental results were divided into two parts. First, the static data results included the relationship between the individual sensors (1 to 5) with the AI as well as the relationship between the force sum of the first three sensors and all five sensors and the AI. Second, the dynamic data results included the relationship between the maximum value of one to five sensors sensing when the subjects walked and the AI.

### 3.1. Static Standing Trials

The Pearson correlation between the forces index calculated from the force sensors during the static and dynamic trial (with different sensors combination) with the ink-type AI value is shown in Table 2. The highest correlation was found on the sensors at the medial arch position (sensor 5 + 4 + 3/2) with the Pearson correlation coefficient equal to 0.715. Meanwhile, the lowest correlation was found on Sensor 5 with r = 0.0707. The comparison between normal and flatfoot in the static trial is shown in Table 3. There is a significant difference between the normal and flatfoot force value in most of the sensor placement positions.

### 3.2. Dynamic Trials

The Pearson correlation between the maximum force index of the force sensor in the dynamic test and the ink-type AI value is shown in Table 4. The highest correlation in the dynamic trial was found on Sensor 5 with r = 0.801 while the less correlated sensor position in the dynamic trial was found in the Sensor 1 position with r = 0.063. The comparison of the normal and flatfoot pressure value and Mann–Whitney U test results in the dynamic trial is shown in Table 5. There was a significant difference between the normal and flatfoot pressure value in six of the sensor configurations: Sensor 3, Sensor 4, Sensor 5, 3 Sensor point (1, 3, 5), the medial arch sensor position, and the lateral arch sensor position.

**Figure 8:**
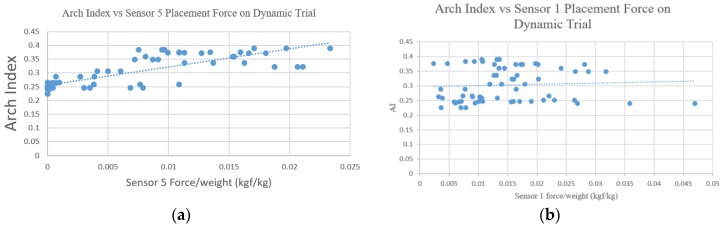
Arch index vs. sensor placement normalized force index in the dynamic trial. (**a**) The best correlation, which was obtained from the placement of Sensor 5; and (**b**) the worst correlation, which was obtained from the placement of Sensor 1.

The linear regression result from the AI vs. Sensor 5 placement in the dynamic trial is shown in Figure 8a. Those results had the highest correlation with r = 0.801 in the dynamic trial. Figure 8b also showed the worst correlation, which was obtained from the AI vs. Sensor 1 placement in the dynamic trial.

Reliability analysis for the system measuring different trials on the same subject was done with an ICC two-way mixed model and Cronbach’s alpha coefficient. The result showed that the proposed measurement system had high reliability for measuring different trials on the same subject with an ICC = 0.812 and Cronbach’s alpha = 0.93.

## 4. Discussion

### 4.1. Placements of the Sensors

To the best of our knowledge, the majority of previous papers have suggested the placement of the force sensitive resistance (FSR) for some specific reference sites of the foot itself, such as the lateral arch and forefoot [18,21]. The current study proposed a more general guideline for the placement of the FSR relative to a given anatomy site, like the commonly targeted medial arch taken in the current study, by describing the placement site in terms of percentage of the insole along the length of the insole itself, which copes with the size of the participant’s foot. The established FSR placement indication on the insole could also help provide guidelines regarding the placements of the FSR from a single one to numerous ones according to the needs for clinical application during the current and future development of the prototype. Based on this repeatability consideration, the author could then perform the human testing that aimed to first perform the correlation between traditional AI and the value of each point, which is a new way to define the placement of the sensor in this study to find out the position of the effective plantar pressure sensor. In addition, unlike the aims of those previous research and development studies reported in the literature, the current study aimed to compare whether there were significantly different values obtained from sensors at specific positions between the flatfoot and normal foot using the data of 21 young subjects.

### 4.2. Static Trials

The results of the static standing trial showed that there was a high linear correlation between the medial arch sensor (5 + 4 + 3/2) with the AI obtained from the static ink (r > 0.7). Meanwhile, five sensor configurations (sensor 1, sensor 3, 3-sensors point (1, 3, 5), 5-sensors point (1–5), and the lateral arch sensor (1 + 2 + 3/2) showed a moderate correlation with 0.4 < r < 0.6.

The ink-type AI has been reported to also be correlated with the navicular height [13,25], the medial arch angle analyzed by X-ray [14,26,27], and the pressure of the hindfoot [28,29,30] and midfoot [31,32] measured during gait. The results of the current study indicated that the force value measured by the insole-type sensor alone was also associated with the AI measured by the traditional ink one, which may be introduced as another method to distinguish between a flatfoot and normal foot. Although significant differences between normal foot and flatfoot were also found (*p* < 0.05), the current method should be applied with caution for individuals whose AI value falls on the transition area (from normal to flat foot).

The results showed that the force of the center midfoot of the flatfoot was obviously higher than the normal foot, which was consistent with the results of the current study. There were obvious differences between the medial arch of the flatfoot and normal foot. Thus, in the static experiment, it was found that the sensors on the arch could be placed in the position of 3 or 5 for the instruments to judge between flatfoot and common foot in the static design. There was a high linear correlation with the AI from the conventional ink-type measurement, and the comparison between the groups showed that there was a significant difference between the sensing measurements of the position of 3 or 5 when judging between normal foot and flatfoot (*p* < 0.05).

### 4.3. Dynamic Trials

The results of the dynamic walking trials pointed out that the medial sensor and Sensor 5 and the medial arch sensor position had high linear correlations (r > 0.7) with the ink-type AI, and different calculation methods were designed mainly with MLA. The linear correlation between Sensor 4 and MLA was merely moderate, speculated to be the same as the results observed when static.

For the comparison of data measured in the walking trials between the flatfoot and normal foot, the calculated force sum of five sensors, three forefoot arch sensors or forefoot arch and Sensor 3, Sensor 4, and Sensor 5, all had significant differences (*p* < 0.05). The force in the midfoot of the flatfoot was greater than that of the normal foot, suggesting the drop of flatfoot arch may result in a poor buffer or propulsive mechanism, as is what has been reported regarding the alternation of the peak value of the ground reaction force between the flatfoot and normal foot population. Without using an expensive force plate to measure the ground reaction force, the results of the pressure sensing test reported in the current study can also provide evidence for the significant differences between the medial and lateral arches of the flatfoot and normal feet, which can be further used to distinguish between a flatfoot and normal foot.

There have been various methods and instruments used to diagnose flatfoot in the past few decades [33,34], but there are still no unified methods for determining flatfoot by referring to both the static and dynamic data. Several parameters measured by the smart insole developed in the current study were compared with the AI and proved to be correlated. For better classification of the type of foot arch, Sensor 5 was sufficient to measure whether the foot was flat, which can be another alternative to facilitate and simplify the procedure of assessing the arch of the foot.

### 4.4. Research Limitation and Future Works

The effectiveness of using AI as a measurement of judging the foot has been questioned as the AI of 24 either overweight or obese elders were related to the body mass index (BMI) [35,36] and the AI measurement could reflect the “fatness” of the foot rather than the “flatness” of the foot. As it has been indicated that obesity may influence the middle third of the foot, especially in overweight or obese subjects [37], the BMI should be considered as a potentially confounding factor when comparing differences between groups of the AI.

In terms of sensor placement, it has been suggested that future studies place sensors on the insole in other remote positions from the arch, such as the great toe or the heel, to complete the design concept by developing a sensor map of the entire foot with more index obtained from different combinations of measured pressure. The guideline of the placements of the sensors in the current study could serve as one of the options for future studies or for future smart insole development that involves the monitoring of the arch-relative index in the industry.

In terms of the algorithm, it is suggested that an artificial intelligence algorithm with a mass of available data is applied to find out the importance of each sensing point in the application to improve the efficiency and accuracy of judgment. As correlations were noted between the AI and musculoskeletal diseases in previous studies, such as plantar fasciitis [38], midfoot osteoarthritis [39], and medial knee osteoarthritis [37], the application of the current method to a variety of clinical patients is also suggested in future studies.

## 5. Conclusions

An Arduino-based pressure-sensor insole was prototyped for assessing flatfoot by choosing a suitable software and hardware architecture from measuring to receiving and designing a simplified self-made measurement plantar pressure instrument. After defining the position of the arch sensors relative to the length of the insole, which served as the placement rule of the sensors at the arch, the effectiveness of different sensor placements and flatfoot type assessment were tested during static and dynamic trials. By performing a correlation between traditional AI and the value of the self-made insole-based sensors with different sensor-placement options, and by comparing whether there were significant differences between the sensors at specific positions of flatfoot and normal foot from the data of 21 young subjects, it was found that during both the static and dynamic tests, the self-developed prototype could determine most of the flatfoot subjects and the most effective plantar pressure sensor position was also determined. Thus, in addition to the traditional subjective judgment of doctors, expensive X-ray detection, or judgments using a conventional ink-type area, the low-price insole-type measurement, including both static standing and dynamic waking, testing could be another wearable tool that reflects the medial longitude arch of the foot. It is suggested that in the future, this device can be applied in the insoles to examine the AI of the foot in clinical purposes due to its advantages when compared to the expensive-high tech graphic input board and conventional tools.

## Figures and Tables

**Table 1:** Subject characteristics of 21 young subjects who participated in this study.

Subject Information	Normal Foot (N = 11)		Flatfoot (N = 10)	
	Mean	±Std	Mean	±Std
Age (year)	23.4	1.0	23.1	2.2
Height (cm)	176.6	3.0	173.1	5.6
Weight (kg)	70.5	6.9	72.2	7.8
BMI (kg/m^2^)	22.6	2.1	24.0	2.5
Arch Index	0.251	0.016	0.356	0.027
Shoe Size (US Size)	9.6	0.4	9.5	0.43
Ratio of foot length and navicular-to-toe length	0.63	0.01	0.64	0.01

**Table 2:** Pearson correlation between the force sensor standing value and the ink-type AI value in the static test.

Parameters			r	*p* Value
	Mean (kgf/kg)	Standard Deviation		
Sensor 1	0.00688	0.00424	0.406	0.001 **
Sensor 2	0.01081	0.00346	0.336	0.008 *
Sensor 3	0.00602	0.00397	0.678	0.000 **
Sensor 4	0.01562	0.0045	0.417	0.001 **
Sensor 5	0.00339	0.00471	0.0707	0.000 **
3 Sensors Point (1, 3, 5)	0.01629	0.01138	0.680	0.000 **
5 Sensors Point (1–5)	0.04272	0.01613	0.668	0.000 **
Medial Arch (5 + 4 + 3/2)	0.02202	0.00917	0.715	0.000 **
Lateral Arch (1 + 2 + 3/2)	0.02069	0.00868	0.487	0.000 **

** Correlation was significant at the 0.005 level (two-tailed).

**Table 3:** Comparison of normal and flatfoot pressure value in the static trials.

Parameters	Normal Foot		Flatfoot		*p* Value
	Mean	Standard Deviation	Mean	Standard Deviation	
Sensor 1	0.00462	0.00205	0.00929	0.00456	0.009 *
Sensor 2	0.00931	0.00261	0.0124	0.00347	0.029 *
Sensor 3	0.00342	0.00108	0.0088	0.00397	0.003 **
Sensor 4	0.01371	0.00469	0.01765	0.00311	0.049 *
Sensor 5	0.00024	0.00059	0.00676	0.00477	0.000 **
3 Sensors Point (1, 3, 5)	0.00827	0.00223	0.02485	0.01078	0.000 **
5 Sensors Point (1–5)	0.03129	0.00648	0.05491	0.01401	0.001 **
Medial Arch (5 + 4 + 3/2)	0.01566	0.00519	0.02881	0.00728	0.000 **
Lateral Arch (1 + 2 + 3/2)	0.01563	0.00412	0.02609	0.00886	0.006 **

* *p* < 0.05, significant difference, ** *p* < 0.005, significant difference.

**Table 4:** Pearson correlation between the force sensor standing value and the ink-type AI value in the dynamic test.

Parameters			r	*p* Value
	Mean (kgf/kg)	Standard Deviation		
Sensor 1	0.03655	0.00861	0.063	0.627
Sensor 2	0.03127	0.00543	0.343	0.006 *
Sensor 3	0.02922	0.00692	0.541	0.000 **
Sensor 4	0.04123	0.00747	0.505	0.000 **
Sensor 5	0.02017	0.00695	0.801	0.000 **
3 Sensors Point (1, 3, 5)	0.056	0.01529	0.604	0.000 **
5 Sensors Point (1–5)	0.11451	0.02282	0.587	0.000 **
Medial Arch (5 + 4 + 3/2)	0.06643	0.01319	0.784	0.000 **
Lateral Arch (1 + 2 + 3/2)	0.06069	0.01374	0.319	0.011 *

** Correlation was significant at the 0.005 level (two-tailed).

**Table 5:** Comparison of the normal and flatfoot pressure value in the dynamic trials.

Parameters	Normal Foot		Flatfoot		*p* Value
	Mean	Standard Deviation	Mean	Standard Deviation	
Sensor 1	0.01351	0.00859	0.01605	0.00555	0.105
Sensor 2	0.01883	0.00374	0.02223	0.00529	0.139
Sensor 3	0.01104	0.00421	0.01798	0.00633	0.007 *
Sensor 4	0.02304	0.00727	0.02906	0.00558	0.035 *
Sensor 5	0.00168	0.00249	0.01291	0.00466	0.000 **
3 Sensors Point (1, 3, 5)	0.02574	0.00967	0.04532	0.01071	0.000 **
5 Sensors Point (1–5)	0.06191	0.01307	0.08878	0.01926	0.067
Medial Arch (5 + 4 + 3/2)	0.02812	0.00758	0.04735	0.00912	0.001 **
Lateral Arch (1 + 2 + 3/2)	0.03461	0.0111	0.04401	0.00961	0.006 *

* *p* < 0.05, significant difference; group effects were analyzed using the Mann–Whitney U test.
